# Counterbalancing the time-dependent effect on the human mitochondrial DNA molecular clock

**DOI:** 10.1186/s12862-020-01640-5

**Published:** 2020-06-29

**Authors:** Vicente M. Cabrera

**Affiliations:** Departamento de Genética, Universidad de La Laguna, E-38271 La Laguna, Tenerife, Spain

**Keywords:** Human evolution, Molecular clock, Mutation rate

## Abstract

**Background:**

The molecular clock is an important genetic tool for estimating evolutionary timescales. However, the detection of a time-dependent effect on substitution rate estimates complicates its application. It has been suggested that demographic processes could be the main cause of this confounding effect. In the present study, I propose a new algorithm for estimating the coalescent age of phylogenetically related sequences, taking into account the observed time-dependent effect on the molecular rate detected by others.

**Results:**

By applying this method to real human mitochondrial DNA trees with shallow and deep topologies, I obtained significantly older molecular ages for the main events of human evolution than were previously estimated. These ages are in close agreement with the most recent archaeological and paleontological records favoring the emergence of early anatomically modern humans in Africa 315 ± 34 thousand years ago (kya) and the presence of recent modern humans outside of Africa as early as 174 ± 48 thousand years ago. Furthermore, during the implementation process, I demonstrated that in a population with fluctuating sizes, the probability of fixation of a new neutral mutant depends on the effective population size, which is in better accordance with the fact that under the neutral theory of molecular evolution, the fate of a molecular mutation is mainly determined by random drift.

**Conclusions:**

I suggest that the demographic history of populations has a more decisive effect than purifying selection and/or mutational saturation on the time-dependent effect observed for the substitution rate, and I propose a new method that corrects for this effect.

## Background

During the last three decades, mitochondrial DNA (mtDNA) variation has played a dominant role in studies of human evolution. Recently, the analysis of this small molecule has been substituted by the analysis of whole genomes. However, before completely turning the page on this type of analysis, it would be convenient to solve the patent contradictions between mtDNA molecular clock time estimations and those recently proposed on the basis of paleontological and archaeological data.

A key achievement of early mtDNA analyses was the dating and origin of the most recent common female ancestor of all living women to approximately 200 kya in Africa [1]. However, hominin fossils and associated Middle Stone Age artifacts from Jebel Irhoud in Morocco have recently been aged to 315 ± 34 kya [2]. These older dates were genetically confirmed in a study of ancient African genomes that estimated modern human divergence to have occurred at 350 to 260 kya [3].

Another controversial milestone of the mtDNA molecular clock is the dating of the dispersal of modern humans out of Africa to 50 to 70 kya based on the coalescence age of macrohaplogroup L3 [4]. This timing is not in agreement with the presence of early modern human remains in the Levant at the Skhul and Qafzeh caves dated to approximately 80–120 kya [5], the presence of middle stone age industries on the Arabian peninsula with similar dates of approximately 80–130 kya [6–8], the recent discovery of unequivocally modern human teeth dated to 80–120 kya in southern China [9], or the recently reported detection of ancient gene flow from early modern humans to the ancestors of eastern Neanderthals more than 100 kya [10]. Furthermore, the most recent discovery of a *Homo sapiens* maxilla at Misliya Cave, Israel, dated to 177–194 kya [11], could significantly anticipate the exit of *Homo sapiens* from Africa. Curiously, these dates within the last interstadial of marine isotope stage 7 (MIS-7) are in agreement with the age proposed for ancient African hominin introgression into European Neanderthals [12].

On the other hand, the optically stimulated luminescence (OSL) dating of stratigraphic undisturbed basal stone tool assemblages in Madjedbebe [13] placed the human colonization of Australia at approximately 65 kya with minor age uncertainties of only ±3–4 kyr. The above date is significantly older than the 43–47 kya coalescence age recently estimated from Australian aboriginal mitogenomes [14].

Under the actual molecular rate estimates given for different sequences of the human genome and the accepted constancy of the molecular clock over time, all of these older archaeological and fossil dates are evidently in conflict with analogous molecular estimates. Initially, the molecular coalescence ages were calculated by means of simple statistics such as the still popular rho statistic [15], based on the average number of polymorphisms observed in a set of related sequences. Subsequently, more sophisticated Bayesian-based methods using relaxed clock phylogenies have been implemented [16]. However, the ages obtained by applying these simple and complex methods to several outstanding events in human history have provided similar age timeframes for them [17]. Thus, it could be that all the above mentioned old human dispersals, detected from the archaeological record represent failed dispersals that did not contribute to the present-day genetic pool of modern humans or that the molecular clock approach is needed for additional adjustment of these estimates.

In this paper, I try to demonstrate that under neutral molecular theory conditions using an overall mitogenome germ line mutation rate and taking into account past fluctuations in the effective population size deduced from any tree topology, it is possible to obtain slower molecular substitution rates that are more in frame with these fossil-based calibrations.

## Results

### Application of the new rho statistic to the main events in human evolution

To apply the new rho statistic described in the Methods section to real human mtDNA data, a rooted tree showing the relationships of the sampled sequences is necessary. Using coalescent methodology, we could obtain a probabilistic tree. However, in the case of human mtDNA, we have a very contrasted phylogenetic tree [18] constructed using the Network program [19]. In this tree, mutations are placed hierarchically from the tips to the roots, and multiple hits identified by network reticulations have been resolved according to the relative mutation rate of the positions involved [17]. Thus, following this standard, I constructed an African mtDNA genome-based phylogenetic tree using 86 previously published complete mtDNA sequences in which all the main African haplogroups are represented (Figure S1). Likewise, using 142 published complete mtDNA sequences, I constructed a second phylogenetic tree for Australasian-specific haplogroup P (Figure S2). Finally, to test a more recent human colonization, I constructed a third tree including 48 already published complete mtDNA sequences belonging to the Americas-specific haplogroup B2 (Figure S3). Using these trees, I applied the proposed time-dependent-based estimator to calculate the coalescence ages of several essential nodes in human history (Tables S1, S2, S3, S4, S5, S6, S7, S8, S9, S10, S11).

I found a TMRCA for all the extant human African mtDNAs of 315,801 ± 17,827 years (Table 1 and Table S2), which is highly compatible with the recent archaeological and paleontological estimations of modern human origin of approximately 315,000 ya [2, 20].

**Table 1:** Coalescence age estimates for several human mtDNA-based evolutionary events

Haplogroup split	Evolutionary event	Mean age in years	95% Coefficient interval
L0/L1’2′5’6′4’3	Most recent African common ancestor	317,814 ya	(352755–282,873 ya)
L3’4	Out of Africa	165,610 ya	(190833–140,387 ya)
L3	Return to Africa of L3	112,829 ya	(133648–92,010 ya)
P	Reaching the Pacific	106,752 ya	(127003–86,501 ya)
P	Reaching Australia	108,034 ya	(128406–87,662 ya)
P	Reaching Philippines	111,545 ya	(132244–90,846 ya)
P	Reaching New Guinea	112,070 ya	(132818–91,322 ya)
B2	Expansion Americas	37,701 ya	(49735–25,667 ya)

Recently, an early out-of-Africa hypothesis for modern humans carrying haplogroup L3 precursor lineages within a favorable time window of approximately 125,000 ya was proposed [21], which is in accord with the age calculated here for the L3’4 split in Africa of 165,610 ± 12,869 ya, as a lower boundary (Table 1 and Table S3). Furthermore, this age timeframe is compatible with the presence of modern humans in the Levant [5] and in China [22] at approximately 100,000 ya.

In the same paper, a return to Africa of basal L3 lineages over 75 kya was also suggested. Again, the age calculated for the L3 African expansion with the method reported here of 112,829 ± 10,622 ya makes this suggestion feasible (Table 1 and Table S4). Furthermore, under this new temporal window, the great morphological variability of the Skhul/Qafzeh remains, and the corresponding wide range of ages (120–80 kya) could easily fit into the whole molecular period proposed elsewhere [21], beginning with the out-of-Africa expansion of early modern humans and ending with their early return to the same continent carrying basic L3 lineages (125–75 kya). However, it should be noted that a return to Africa from the Arabian Peninsula would also be supported by the dates estimated from the archaeological record of the region.

On the other hand, our TMRCA for Australasian haplogroup P (103 267 ± 10,332 ya) was also in agreement with an early presence of modern humans in Asia (Table 1 and Table S6, Table S7, Table S8, Table S9). Thus, this timing indicates a lower boundary for the colonization of the Philippines [23] Sumatra [24] and Australia [13] of approximately 65,000 to 73,000 ya. It has to be mentioned that from the genome sequencing of an Aboriginal Australian [25], it was deduced that Aboriginal Australians are descendants of human dispersal into Eastern Asia that occurred approximately 62–75 kya.

Finally, the time of human expansion to the American Continent deduced from the haplogroup B2 phylogeny was approximately 37,000 ya (Table 1, and Table S11). This age supports a pre-Clovis occupation of the New World, well before the last glacial maximum.

## Discussion

The mtDNA ages calculated for the main human movements out of Africa applying the method proposed here are in remarkable concordance with those obtained from the most recent paleontological and archaeological records, which were conversely in open conflict with the hitherto most recent mtDNA molecular data [26]. However, as the ages calculated here have wide statistical confidence intervals (Table 1), different models could be adjusted to fall within their timeframes. For example, we might assume an earlier out-of-Africa expansion matching the Misliya maxilla dated to 177–194 kya; then, the Skhul and Qafzeh remains dated to approximately 80–130 kya might signal the return to Africa of the carriers of the basal mtDNA haplogroup L3 lineages, rather than the out-of-Africa expansion of early anatomically modern humans as proposed here. Similarly, the controversial presence of *H. sapiens* in Java [27] and Sulawesi [28] as early as 120 kya would fit into the age window of Australasian mtDNA haplogroup P (Table 1). Future archaeological discoveries and more precise fossil dating will help to determine the most appropriate model.

It is worth mentioning that a return to Africa of carriers of the basal maternal L3 and paternal E lineages, as we proposed previously [21], dated here to approximately 75 kya, has received strong support from a recent study that provides evidence of Neanderthal sequences present in modern Africans, most likely as the result of the early back-migration of putative Eurasian groups to Africa [29]. Interestingly, there is a possibility of testing whether these Eurasian groups were effectively carriers of the mtDNA L3 and Y-chromosome E lineages because, were this the case, native African groups such as the eastern African Hadza and Sandawe, the southern African Khoesan and the central African pygmies should exhibit comparatively less Neanderthal ancestry introgressed into their genomes than the group that returned to Africa later.

On the other hand, there is also recently published archaeological evidence that shows that modern humans could have colonized the central Siberian Arctic as early as 45 kya [30]. This gives rise to the possibility of an earlier entry into the Americas, as proposed here.

Finally, one must be aware that in addition to its wide uncertainty range, the method proposed here depends on the accuracy of several external assumptions. One is the overall germline mtDNA mutation rate estimated for the studied species. For example, after this article was written, a new paper addressing the germline mtDNA variability within humans with an experimental design as rigorous as that used by Rebolledo-Jaramillo et al. [31] was published [32]. The authors estimated the overall germline mtDNA mutation rate per site per generation to be 4.72 × 10^− 7^ (95% bootstrap CI: 3.93–5.52 × 10^− 7^), which was approximately 75% higher than the rate used here. Thus, it would be convenient if, when more empirical estimates accumulated, a consensus average rate could be reached. This can also be extended to the average human generation interval, which is necessary to convert generations into years.

Additionally, this algorithm is highly dependent on the degree to which the tree reflects the demographic history of the analyzed population and therefore on the demographic parameters assumed in the construction of the tree for either phylogenetic [33] or coalescent methods [34].

To a lesser degree, this algorithm is also sensitive to the sample size used to construct the tree to identify nodes that include less frequent lineages that are still present in the extant population. Fortunately, in the case of humans, mtDNA diversity has been exhaustively sampled at both the continental and population levels in recent decades, so the risk of missing rare lineages is very low.

## Conclusions

Taking into account the time dependence of the mtDNA evolutionary rate in humans as proposed here and choosing a conservative mtDNA germ-line mutation rate, as experimentally obtained by others [31], has resulted in a significantly slower mtDNA molecular clock in humans in such a way that all the main events of human history dated by paleontological and archaeological methods fit within this new mtDNA temporal scale without the necessity of external node calibrations. Finally, it should be stressed that in my opinion, the applicability of this method to other demographic scenarios and other markers susceptible to being represented in phylogenetic trees, such as the Y-chromosome and the nonrecombining portions of the autosomes, deserves further investigation.

## Methods

### The applied human mitochondrial mutation rate

The efficiency of the molecular clock [35] is based on the reliability of several implicit assumptions, such as a) the correctness of the mutation rate point estimate (μ) of the gene under study; b) the constancy with time of the rate of molecular substitution (Ɵ); and c) the rate homogeneity among the different lineages involved in the phylogeny. To address the first point, in this study, I used the full-length mtDNA germ-line mutation rate of 1.3 × 10^− 8^ (interquartile range, 4.2 × 10^− 9^ to 4.1 × 10^− 8^) mutations per site per year (assuming a generation time of 20 years) and its derived rate scalar of one mutation every 4651 years estimated by others [31]. This mtDNA mutation rate is approximately ten times lower than the estimates in most pedigree studies, which the authors explain by their approach of the analysis of two tissues, which allowed them to discard somatic heteroplasmies. In this respect, it must be mentioned that second-generation massive sequencing has made possible the direct calculation of the human germline genomic mutation rate, which reduced the phylogenetic mutation rate by half, thus doubling the estimated divergence dates of Africans and suggesting that crucial events in human evolution occurred earlier than suggested previously [36].

### Accounting for the time-dependent effect on the rate of molecular evolution

It is well established that rates of molecular evolution are not constant at interspecific or intraspecific levels [37]. In general, they decline with increasing divergence time, but the rate of decay differs among taxa. This time-dependent pattern has also been observed for human mtDNA in both coding and noncoding regions [38, 39]. Purifying selection on deleterious mutations and mutation saturation have been suggested as the main forces responsible for this time rate decay [39]. However, the unrealistic large effective population sizes required to explain the long-term persistence of significantly deleterious mutations cast doubt on whether purifying selection alone can explain the observed rate acceleration [40]. It has also been found to be unlikely that the apparent decline in rates over time is due to mutational saturation [39]. Congruently, correcting for the effects of purifying selection and saturation has only slightly modified the mtDNA evolutionary mutation rate, providing molecular times that are still in apparent contradiction with archaeological and paleontological ages [17, 39]. Demographic processes such as serial bottlenecks and expansions have also been proposed to explain the differences in rate estimates over time [41]. It seems evident that some adjustment should be implemented to correct the time dependency of the molecular clock. In this paper, I propose a practical approach for counteracting the time-dependent effect on molecular rate estimates, taking into account tree topologies.

### Approaching the lack of mutation rate homogeneity between lineages

Since some of the earliest molecular analyses, it has been observed that rates of homologous nuclear DNA sequence evolution differ between taxonomic groups [42], which can be extended to mtDNA [43]. Later, significant differences in the rates of molecular evolution between mtDNA human lineages were also detected at the haplogroup level [44–48]. Different relaxed molecular-clock methods have been implemented to incorporate rate variation among lineages [49, 50]. However, the application of these methods to human mtDNA has yielded age estimates for the main milestones of human evolution that are in agreement with previous molecular estimates [51, 52]. In this paper, when distributing the mutations of lineages with significant rate differences within coalescent periods, I used a simple proportionality criterion. I allowed a window of 0 to 5 mutations between sequences within coalescent periods, as it has been demonstrated that under a Poisson distribution, even over an extended period of 10,000 years, lineages that still carry the same mutations as their common ancestor could still exist, in addition to lineages that have accumulated five new mutations, with probabilities higher than 0.05% [53]. Another technical problem is the mutation distribution among coalescent periods of the isolated sequences that directly radiate from ancestral nodes. To resolve this issue, I used a weighted distribution calculated by multiplying the number of mutations in the isolates by the number of mutations between internodes in each coalescent period and then dividing the result by the total number of mutations at all the internodes.

### Effect of fluctuating population size on mutation substitution rates

Within a population, the fixation time of a mutation (forward) or the coalescence time to the most recent common ancestor (backward) is usually calculated by the estimator Ɵ = 4N_e_μ (where N_e_ is the effective population size). For the haploid mtDNA genome, Ɵ equals 2N_ef_μ (being N_ef_ the female effective population size). Kimura demonstrated that under strict neutral theory parameters, the rate of substitution is equated to the mutation rate [54]. However, the same author warned us that a clear distinction exists between the mutation rate (μ) and mutation substitution (Ɵ). The former refers to the change in genetic material at the individual level, and the latter refers to that at the population level [54]. Thus, only when N_e_ is constant across generations, maintaining small or large sizes, is Ɵ equal to μ. This holds because with large constant sizes, the number of new mutations incorporated into the population (Neμ) increases, but along the same path, the probability of fixation (1/Ne) decreases. In contrast, with small constant sizes, the number of new mutations decreases, but the probability of the fixation of any of the mutations increases at a similar level. It is widely admitted that N has fluctuated greatly during human history and that global exponential population growth has occurred in recent times [41]. In this paper, I have taken into account the changes in population size that occurred backward in time and their influence on the rate of gene substitution. When the population size fluctuates across generations, the probability of fixation of a neutral mtDNA variant (*q*) is no longer the 1/N constant. It will depend on the difference in the population size of the next generation (N_1_) with respect to the initial size (N_0_):

$$ q=\frac{N_1}{N_0}\times \frac{1}{N_0} $$

For example, if N_1_ is twice the size of N_0_, *q* equals 2/N_0,_ and, on the contrary, if N_1_ is half the size of N_0_, *q* equals 1/2N_0_. As a consequence, the rate of substitution for neutral mutations in a population of fluctuating size depends on the change in size between generations:

$$ \uptheta =\frac{N_1}{N_0}\times \mu $$

Using a different approach, the dependence on the population size of the substitution rate at neutral genes was already demonstrated for populations with fluctuating sizes and overlapping generations [55].

As human populations have been growing exponentially for several centuries, we should counterbalance this effect from the present-day generation (N_n_) going backward in time by inverting the fraction between consecutive generations (N_n-1_/N_n_). Note that this dependence might explain the differences in rate estimates over time observed empirically [38]. I will take into consideration this important relationship for the calculation of Ɵ.

### A new rho statistic for estimating coalescent ages

**Fig. 1:**
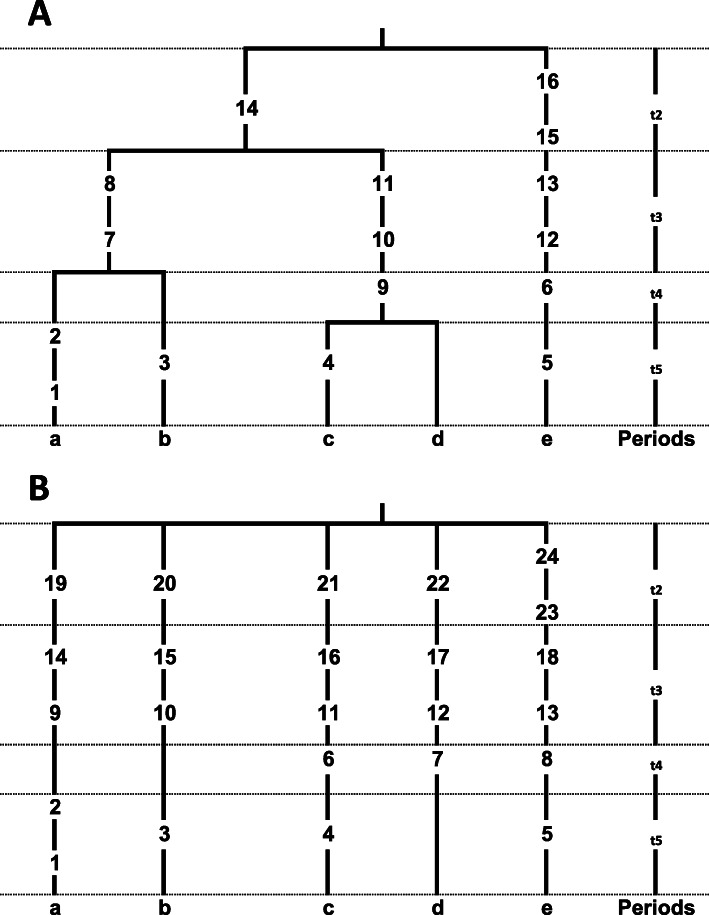
**a** Empirical coalescent tree of five lineages (a, to e) with four coalescent periods (t) and mutations along branches (numbers). **b** Ideal star-like tree for the same five lineages

Several statistics based on DNA polymorphism exist for estimating the parameter Ɵ. One such statistic, S, the number of segregating sites per nucleotide in a sample of sequences [56], is strongly dependent on the sample size. A second, π, is defined as the average number of nucleotide differences per site in a sample of sequences [57]. These two estimators were used to implement a statistical method for testing the neutral mutation hypothesis [58]. A third statistic, rho (ρ), is referred to as the mean number of nucleotide differences in a sample of sequences compared to their common ancestral type [15]. This last statistic is calculated from a rooted phylogenetic tree showing the relationships of the sampled sequences. The accuracy of molecular dating with the rho statistic has been questioned by some because it shows downward biased data estimations, large asymmetric variances and strong dependency of demographic factors [59], but it is defended by others [60]. Regardless, it is still a commonly used method for measuring intraspecific mtDNA divergence events in humans. Although the distribution of pairwise nucleotide site differences between individuals has been used to detect episodes of population growth and decline [61], none of the abovementioned statistics considers the past demography of the sample in their age estimates. In this paper, I propose the use of a modified rho (ρ_m_) that by taking into account the coalescent genealogical structure, significantly improves molecular date estimation for key events in human history based on mtDNA genome data. To make explicit our modifications to the classical rho, I have depicted a real genealogy constructed from five lineages (a) and an idealized star-like phylogeny of the same five lineages (b) in Fig. 1, assuming population exponential growth shortly after a severe bottleneck [62]. The number of lineages sampled is represented by n_i_; t_i_ represents the time periods defined by progressive coalescent events from the tips to the most recent common ancestor (TMRCA) root; i represents the number of independent lineages remaining after successive coalescences; ϒ_i_ is the number of mutations accumulated during each coalescent period; and m_i_ is the number of mutations accumulated along each lineage. Mutations in the star-like phylogeny are distributed into periods following the pattern found in the real phylogeny. As the accumulation of mutations along each lineage is an individual process driven by the mutation rate, μ, and distributed as independent Poisson processes, ρ, the average number of mutations per lineage has the same value irrespective of the topology. However, as a consequence of the fact that the lineages in the real tree are not independent because of their shared genealogy, the rate of variance decay is much slower (1/logn) than in the independent star-like tree (1/n) [63]. As a consequence, for the rho calculation, mutations within lineages in the star-like phylogeny are counted only once. In contrast, in the real phylogeny, only mutations occurring at the tips are counted only once, while mutations in subsequent coalescent periods going backward to the MRCA node are counted as many times as the number of periods to which they belong. For this reason, mutations in the older periods are overrepresented in the rho calculation. Because of lineage independence, star-like phylogenies are statistically optimal for calculating ρ and π estimators. Under this topology, as mutations along lineages are counted from the root to the tips in ρ and from tip to tip in π pairwise comparisons, the value of π is twice that of ρ. However, as rho ignores the dependence among lineages existing in the majority of the trees, it is necessary to correct for this dependence. For this reason, I propose a modified rho statistic (ρ_m_) that represents the summation of the classical rho statistics calculated for each coalescent period in the tree:

$$ {\rho}_{\mathrm{m}}={\sum}_{i=2}^n\rho \mathrm{i} $$

This compound Poisson distribution is also Poisson distributed; therefore, the mean and variance are equal, and the standard deviation is the square root of this variance. Thus, uncertainty in the estimates was calculated using the Poisson confidence interval. Another important difference between the real and star-like genealogies is that in the former, the number of lineages decreases as one stepwise function across coalescent periods, while in the latter, the number of lineages is constant until the root is reached. Equating the number of lineages in the sample as an approximation of the effective population size in the population, we should take into account this backward real decrease in N_e_ to improve the estimation of the MRCA age. In an ideal coalescent model, we should have i-1 decreasing population sizes, but in real phylogenies, in addition to bifurcations, there are also multifurcations and lineages with long internal segments without any branching events. Even so, I applied the reverse proportion used to counteract the time-dependent effect on the evolutionary rate to each rho in consecutive periods going backward. That is, the mutation rate, μ, was multiplied in each i-1 period by (i-1)/i, leaving μ as calculated from the germline estimations for the most recent period, comprising the tips of all the lineages sampled. With this method, I obtained a time-dependent scaled mutation rate, Ɵ, that produced human mtDNA intraspecific ages congruent with the archaeologically and paleontologically calibrated nodes representing key events in human history.

$$ \uptheta =\frac{1}{\upmu}\left({\rho}_n+{\sum}_{i=2}^n\frac{i+1}{i}\cdot {\uprho}_{\mathrm{i}}\right) $$

By performing calculations on the basis of the empirical genealogy (Fig. 1a), I obtained an age of 22,277 ± 4720 years using the standard ρ and an age of 30,396 ± 5513 years (1.36 times greater) when using the time-dependent Ɵ estimator proposed here (Table 2).

**Table 2:** Coalescence age for the Tree in Fig. 1 using the compound rho

Period	Lineages	Mutations	Rho	i/i + 1	μ	1/μ	Years
2	2	3	1.50	0.67	1.44 × 10–4	6944	10,416
3	3	6	2.00	0.75	1.61 × 10–4	6211	12,422
4	4	2	0.50	0.80	1.72 × 10–4	5814	2907
5	5	5	1.00	1.00	2.15 × 10–4	4651	4651

Coalescence age for Fig. 1 tree: 30396 ± 5513

## Supplementary information

**Additional file 1: **Supplementary figures: **Figure S1**. Phylogenetic tree of mtDNA macrohaplogroup L complete African sequences produced in this study. **Figure S2**. MtDNA haplogroup P phylogeny. **Figure S3**. MtDNA haplogroup B2 phylogeny.

**Additional file 2 **Supplementary tables: **Table S1**. Data from Figure S1 mtDNA African tree to calculate the human most recent common ancestor (TMRCA). **Table S2.** Age of the African mtDNA most recent common ancestor. **Table S3**. Age of the mtDNA haplogroup L3’4 split. **Table S4**. Age of the mtDNA L3 African expansion. **Table S5**. Data from Figure S2 mtDNA haplogroup P tree to calculate the Pacific human most recent common ancestor (TMRCA). **Table S6**. Age of the Pacific mtDNA haplogroup P most recent common ancestor. **Table S7**. Age of the mtDNA haplogroup P colonization of Australia. **Table S8**. Age of the mtDNA haplogroup P colonization of the Philippines. **Table S9**. Age of the mtDNA haplogroup P colonization of New Guinea. **Table S10**. Data from Figure S3 mtDNA American haplogroup B2 tree to calculate the Americas colonization age. **Table S11**. Age of the mtDNA haplogroup B2 colonization of the Americas.

## Data Availability

All data analyzed during this study are included in this article and its Supplementary Information files.

## Abbreviations

CI: Coefficient interval
Kya: Thousand years ago
MIS: Marine isotope stage
OSL: Optically stimulated luminescence
TMRCA: The most recent common ancestor
